# A Feasibility Study of SSVEP-Based Passive Training on an Ankle Rehabilitation Robot

**DOI:** 10.1155/2017/6819056

**Published:** 2017-09-17

**Authors:** Xiangfeng Zeng, Guoli Zhu, Lan Yue, Mingming Zhang, Shane Xie

**Affiliations:** ^1^School of Mechanical Science and Engineering, Huazhong University of Science and Technology, Luoyu Road 1037, Wuhan, China; ^2^Department of Mechanical Engineering, University of Auckland, Auckland 1142, New Zealand; ^3^School of Mechanical Engineering, School of Electronic and Electrical Engineering, University of Leeds, Leeds LS2 9JT, UK

## Abstract

**Objective:**

This study aims to establish a steady-state visual evoked potential- (SSVEP-) based passive training protocol on an ankle rehabilitation robot and validate its feasibility.

**Method:**

This paper combines SSVEP signals and the virtual reality circumstance through constructing information transmission loops between brains and ankle robots. The robot can judge motion intentions of subjects and trigger the training when subjects pay their attention on one of the four flickering circles. The virtual reality training circumstance provides real-time visual feedback of ankle rotation.

**Result:**

All five subjects succeeded in conducting ankle training based on the SSVEP-triggered training strategy following their motion intentions. The lowest success rate is 80%, and the highest one is 100%. The lowest information transfer rate (ITR) is 11.5 bits/min when the biggest one of the robots for this proposed training is set as 24 bits/min.

**Conclusion:**

The proposed training strategy is feasible and promising to be combined with a robot for ankle rehabilitation. Future work will focus on adopting more advanced data process techniques to improve the reliability of intention detection and investigating how patients respond to such a training strategy.

## 1. Introduction

Stroke is one of the main root causes leading patients unable to comfortably control their muscles and bodies in the daily living, and even lose the ability [1–3]. The ability of body controlling is inversely proportional to the distance between brains and limbs, which means that the longer the distance is, the lower the ability is [4]. Motor function of injured ankles will be recovered more difficult than one of the hands with a similar disability.

For early stage rehabilitation of injured ankles, if without sufficient rotations, ankle joints could gradually become stiff, and finally, foot drop will be generated [5, 6]. In order to avoid being stiff, muscle stretching and joint rotating are regarded as one of the important methods in traditional therapy of injured ankle joints. Traditional physical therapy is usually operated manually by therapists. It has a unique advantage, which therapists can observe real-time feedback from patients through their body reaction and communication and thus adjust the process accordingly. However, it also has several limitations: (1) therapists can feel weary for long-time operation; (2) operating strength cannot be kept uniformly during the whole process; (3) mental state of therapists is one of the key factors to affect therapy effect [7].

In order to release manpower and address those limitations, robots have been invented to substitute partial functions of traditional therapy [8, 9]. For ankle rehabilitation, there are two kinds of robots invented, one of which is platform-based robots, and the other is wearable devices [3]. When training on platform-based robots, subjects are normally in a sitting position to train their physical function of muscle stretching and joint rotating [8, 10]. When training on wearable ankle robots, subjects are required to be in a standing position to improve their ability on walking [1]. Therefore, platform-based robots can provide better rehabilitation for subjects with weak motion ability of ankle joints, while targeted subjects of wearable ankle robots are those whose motion ability of ankle joints is strong enough to walk, but gait needs to be rebuilt and improved further recovery [11].

Passive training is one of the basic functions of platform-based robots. Different with common passive stretching with constant speed, Zhang et al. [12] proposed an intelligent passive stretching strategy in ankle dorsiflexion/plantarflexion (DF/PF) for safety. During intelligent passive stretching, rotating speed of the robot was inversely proportional to resistance torque. As soon as predefined maximum resistance torque was reached, ankle joints would be held at the extreme position for a period of time to allow stress relaxation. For robot-assisted passive ankle training, subjects are requested to keep relaxed to follow up trajectories of robots [3, 10]. After experiencing passive training, physic function of ankle joints can be kept to a certain degree and foot drop can be alleviated correspondingly [5, 8, 12].

Active training is another function of platform-based robots, where subjects are requested to actuate robots to track targets by allowing the foot to follow visual or auditory instructions [1, 10, 13, 14]. Visual reality circumstance has been widely applied in robot-assisted active ankle training. Girone et al. [15] proposed a virtual reality exercise library on the Rutgers Ankle. Subjects could conduct simulation exercise of strength, flexibility, and balance with haptic and visual feedback. Burdea et al. [14] proposed rehabilitation games including the airplane game and breakout 3D game. Michmizos et al. [16] proposed three goal-directed serious games especially for children. In this study, visual reality circumstance is set as a game of whack-a-mole, which four hamsters are arranged in four directions as targets, and a hammer is initially located in the center as the movable cursor. The vertical trajectory of hammer is projected to DF/PF, while the horizontal one is corresponded to inversion/eversion (INV/EV).

For passive training, subjects do not need to exert active effort, and thus few information transmission loops between brains and ankles exist [17]. A prerequisite of conducting active training is that subjects should have enough motion ability of ankle joints to trigger robots [10]. Therefore, for subjects whose motion intentions of ankle joints cannot be detected by built-in force sensors of robots, solving the problem of how they can actively conduct ankle training is a big challenge. This study aims to construct an information transmission loop between brains and ankle robots and enable subjects with weak motion ability of ankle joints to actively conduct robot-assisted ankle training.

When subjects focus their attention on a flickering source with frequency above 6 Hz, electroencephalography (EEG) signals originated from their visual cortex are named SSVEP, spectrum of which shows peak at the flickering frequency and its harmonics [18]. SSVEP signals have been extracted and applied in many fields, such as controlling the robotic wheelchair [19], the humanoid robot navigation [20, 21], the semiautonomous mobile robotic car operation [22], and the artificial upper limb [23].

In this study, SSVEP signals are introduced and used for passive training on an ankle rehabilitation robot, in which motion intentions of subjects can be extracted to trigger related passive training. Four flickering circles with the diameter of 22 mm are arranged in four directions. Flickering frequencies are set as 10 Hz for the upper, 12 Hz for the bottom, 8.6 Hz for the left, and 15 Hz for the right [24]. For subjects, gazing at the upper flickering circle represents the motion intention for DF, the bottom for PF, the left for INV, and the right for EV.

To enable subjects with weak motion ability of ankle joints to conduct motion intention-directed passive training, this study develops a SSVEP-based passive training strategy through combining SSVEP signals and virtual reality circumstance on an ankle robot. To verify its feasibility, this study recruited five healthy subjects for preliminary evaluation.

## 2. Methods

### 2.1. Ankle Rehabilitation Robot

**Figure 1:**
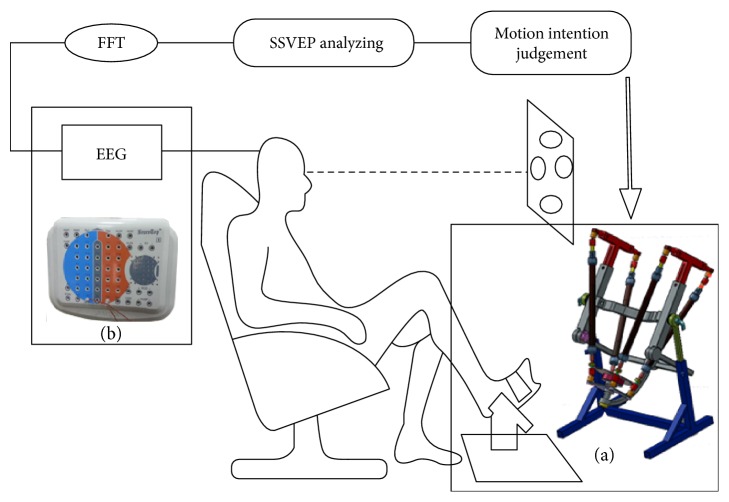
SSVEP-based passive training on an ankle rehabilitation robot. Subjects sit on a comfortable chair and are requested to gaze at one of the flickering circles. Motion intentions are detected through analyzing SSVEP signals. Each detection would immediately trigger the ankle robot to conduct a predefined passive stretching. (a) An ankle rehabilitation robot is applied to provide passive and active ankle training along DF/PF, INV/EV, and AA trajectories. (b) An EEG system (NT9200, symtop) is applied to acquire and amplify EEG signals.

The ankle rehabilitation robot applied in this study is an improved version of the one used in [11] by adding adjustable robot structure and was briefly introduced as in Figure 1(a). The footplate of the ankle robot could move with three degrees of freedom, which are corresponding to ankle DF/PF, INV/EV, and adduction/abduction (AA). The robot is actuated in parallel by four FFMs (FESTO DMSP-20-400N), pressure control of which is regulated by four proportional pressure regulators (FESTO VPPM-6L-L-1-G18-0L6H). Three magnetic rotary encoders (AMS AS5048A) are installed along each axis to measure angular positions forming a three-dimensional coordinate system of the footplate. Four single-axis load cells (FUTEK LCM 300) are installed to measure contraction forces generated by FFMs. A six-axis load cell (SRI M3715C) is installed below the footplate to measure interaction forces and torques between human feet and the footplate.

**Figure 2:**
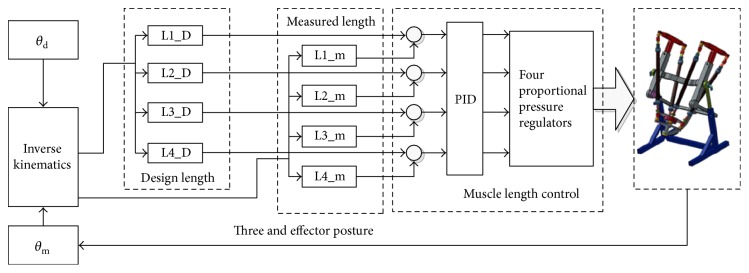
The flowchart of individual muscle length control in joint space.

The position control of this robot can be achieved by controlling individual FFM length in joint space, as shown in Figure 2. The desired individual FFM length is calculated by inverse kinematics based on the desired position of the end effector, while, as the feedback to the PID controller, the actual individual FFM length is obtained by inverse kinematics based on the measured position of the end effector. This joint space position controller outputs four pressure values that directly go to four proportional pressure regulators for the actuation of the robot.

### 2.2. Information Transmission Loop

**Figure 3:**
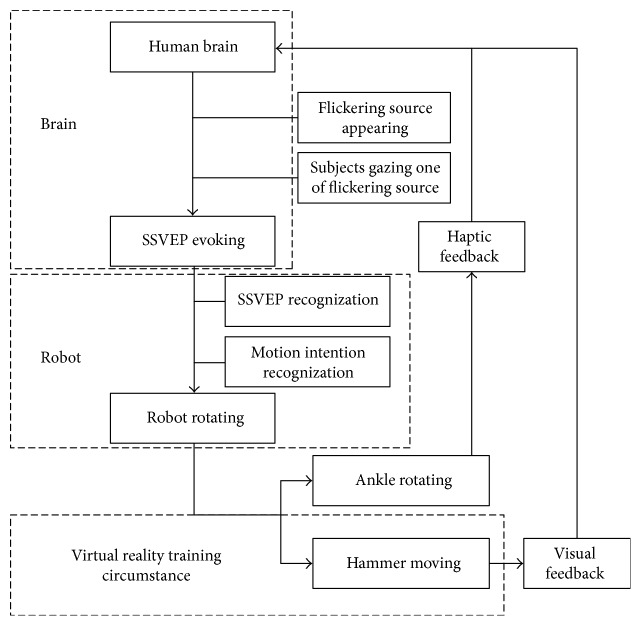
Information transmission loop. SSVEP signals are evoked in brains when subjects pay their attentions to one of the flickering circles. The robot judges motion intentions of subjects through recognizing the flickering source of SSVEP signals and is triggered to deliver predefined training. Haptic feedback ascends to brains when ankles rotate following the robot, and visual feedback of ankle rotation ascends to brains when the hammer moves in virtual reality training circumstance.

**Figure 4:**
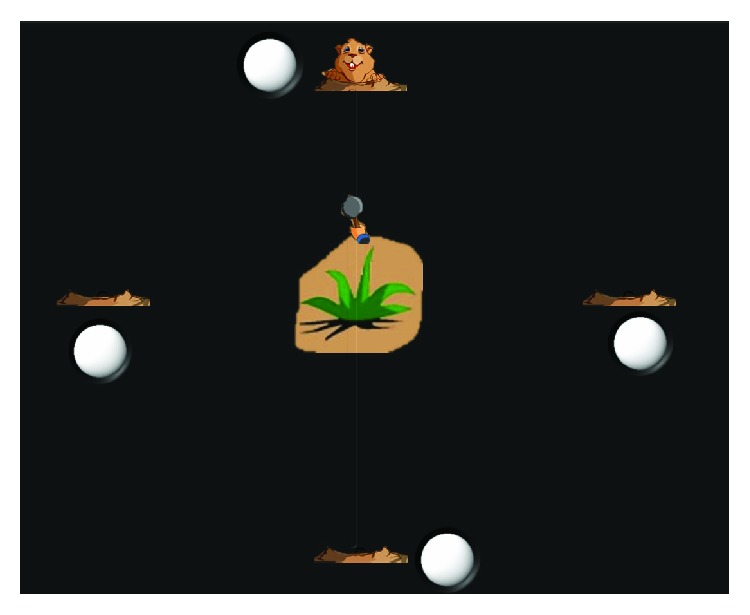
Virtual reality circumstance. Hamsters are set as targets and will disappear when ankle joints rotate to a predefined maximum position. SSVEP signals will be evoked when subjects gaze at one of the flickering circles. Rails of the hammer are mapped to the trajectories of ankle joints.

An information transmission loop between brains and ankle robots is constructed through combining SSVEP signals and virtual reality circumstance for passive training on the ankle rehabilitation robot, as shown in Figure 3. SSVEP signals are evoked in brains when subjects pay their attention on one of the flickering sources, which represents motion intentions of subjects in the training. The robot can recognize motion intentions of subjects by analyzing the flickering source of SSVEP signals and immediately trigger the robot to conduct predefined training. Real-time visual feedback of ankle rotation ascends to brains immediately when the hammer in virtual reality training circumstance moves, as in Figure 4.

#### 2.2.1. Virtual Reality Training Circumstance

The virtual reality training circumstance is set as a game of whack-a-mole, which consists of a black background wall, a vertical rail, a horizontal rail, a hammer, a tent, four Hamsters, and four flickering circles (Figure 4). The vertical rail is projected to DF/PF trajectory, and the horizontal rail is corresponding to INV/EV trajectory. There are four hamsters located at the end of the vertical and horizontal rail, nearby which four circles with a diameter of 22 mm are flickering with frequency of 10 Hz for the upper, 12 Hz for the bottom, 8.6 Hz for the left, and 15 Hz for the right [24]. The tent is located at the cross point between the vertical and horizontal rail, corresponding to the neutral position of ankle joints. The hammer can move freely along with the vertical or horizontal rail, and its position represents the posture of the footplate or the human ankle during the training.

At the beginning of the game, four hamsters and four flickering circles appear on the computer screen, which the upper represents the target for subjects to conduct the training of DF, the bottom for PF, the left for INV, and the right for EV. When subjects focus their attention on one of the flicking circles about 5 seconds, the robot will be triggered to rotate the footplate based on its judgment of motion intention of subjects through analyzing SSVEP signals. Meanwhile, accompanying with the targeted vertical or horizontal rail appearing and the tent disappearing, the hammer will start to move toward the targeted hamster. Once the hammer reaches the end of rails, the targeted hamster will disappear, and then it will return back to the cross point. Tent and hamsters will reappear as soon as the hammer arrives at the cross point, representing that preparation for the next cycle of training is ready.

#### 2.2.2. SSVEP Recognition

EEG signals are acquired and amplified by a video EEG system (NT9200, symtop) that can provide 41 Ag/AgCl electrodes, which are positioned according to international 10/20 system as in Figure 1(b) [25]. Prior to data acquisition, impedance inspection of the EEG system is conducted to verify whether contact resistance among electrodes and scalps can meet design specification after conductive gel is injected to fill up the gap between working electrodes and head of subjects. The signals from Oz is applied to extract motion intentions of subjects, while the reference electrode is placed on earlobe A2 and the ground electrode is placed on Fpz. EEG signals are digitalized and processed through Labview software (National Instruments, Austin, USA), the sampling frequency of which is set up to 500 Hz, and the duration for robot to judge motion intention of subjects is set to 5 seconds. EEG recordings are band-pass filtered from 6 Hz to 30 Hz through the application of the Butterworth filter.

Fast Fourier transform (FFT) [26] is applied to those epochs, which contain 2500 data points for every electrode. According to generation mechanism of SSVEP signals, the peak of amplitude will occur at the flickering frequency and its harmonic when subjects focus their attention on a flickering source [23]. But off-line analysis indicates that it has a slight shift of frequencies for the peak amplitude occasionally, caused possibly by displaying characteristics of LCD. Therefore, it can partially eliminate deviation caused by frequency shift when the maximum amplitude of five adjacent frequencies centered on a flickering frequency is designated as the amplitude of that flickering frequency, which is expressed as in (1), where *X*_*ij*_ is the amplitude of five adjacent frequencies centered on the flickering frequency, *j* is the serial number of five adjacent frequencies, and *i* is the serial number of flickering circles.

(1) $$\begin{array}{c} A_{i}=\max\left(X_{ij}\right),\;\left(i=1,\ldots ,4;j=1,\ldots ,5\right). \end{array}$$

After amplitudes of flickering frequencies are ascertained, the dominant frequency in the SSVEP signals will be identified as the flickering frequency with the maximum amplitude, in (2).

(2) $$\begin{array}{c} f_{target}=\arg\;\max\left(A_{i}\right),\;\left(i=1,\ldots ,4\right). \end{array}$$

### 2.3. Subjects

Five healthy subjects with ages at 24 ± 3 years participated in this study. One of them is female, and others are male. Inclusion criteria are subjects with (i) normal vision, (ii) corrected-to-normal vision, and (iii) no history of clinical visual impairment. Subjects who can be easily distracted will be excluded. All subjects are right-handed. All of subjects are the first time to conduct SSVEP-triggered passive training on the ankle robot. During the training, blinking is not prohibited. The whole experiment was conducted in a laboratory with a floor space of approximately 43 square meter. It is quiet in the surrounding, and light illumination is weak.

### 2.4. Training Protocol

Each subject was requested to sit calmly 50 cm in front of LCD, looking straightly at the virtual reality circumstance, and put the right leg in the ankle rehabilitation robot, with the right foot fixed on the footplate. The electrode cap is placed on the head of subjects following up the regulation of international 10/20 system [25], and electrode gel is applied. Before the training, subjects are informed (i) gazing at the upper flickering circle represents the motion intention for DF, the bottom for PF, the left for INV, and the right for EV; (ii) gazing at the intended flickering circle once the tent appears, and giving up the gazing when the tent disappears or ankle joints begin to rotate; (iii) during stretching of the robot, subjects need to observe moving situation of the hammer and imagine rotation situation of related ankle joints; (iv) if actual stretching of the robot is not consistent with their motion intention, subjects merely follow up the rotation without any resistance against the movement.

Subjects were requested to conduct two kinds of combined ankle movement tasks through focusing on one of the four flicking sources to trigger the robot. One kind of task combines ankle DF training with PF training together, and no time interval exists between them. A total five tasks are set in the training. The other kind of task is the combination of INV and EV training, without time interval among them, and a total of five tasks are set. There is 1 minute for free between both tasks, and SSVEP signals of subjects will not be extracted to judge motion intentions of subjects until the footplate of the robot returns back to the neutral position.

### 2.5. Evaluation Procedures

In this study, the performance of motion intention detection is evaluated by a success rate and information transfer rate (ITR). The success rate is defined as the percentage of output that actual movements of the robot are consistent with motion intentions of subjects. Therefore, the success rate is described as in (3), where *A* denotes the quantity of motion intentions which are correctly recognized and *B* denotes the total quantity of motion intentions which are requested to identify.

(3) $$\begin{array}{c} Success\;Rate=\frac{A}{B}\times 100\%. \end{array}$$

The ITR [27] under the unit of bits/min is expressed as in (4), where *N* is the number of flickering circles, which is set to 4 in this study. *P* is the success rate, and *T* is the time during which SSVEP signals are extracted to determine the motion intention of a subject.

(4) $$\begin{array}{c} B=\left\{\log_{2}N+P{\;\log}_{2}P+\left(1-P\right){\;\log}_{2}\left[\frac{1-P}{N-1}\right]\right\}\times \frac{60}{T}. \end{array}$$

## 3. Results

**Figure 5:**
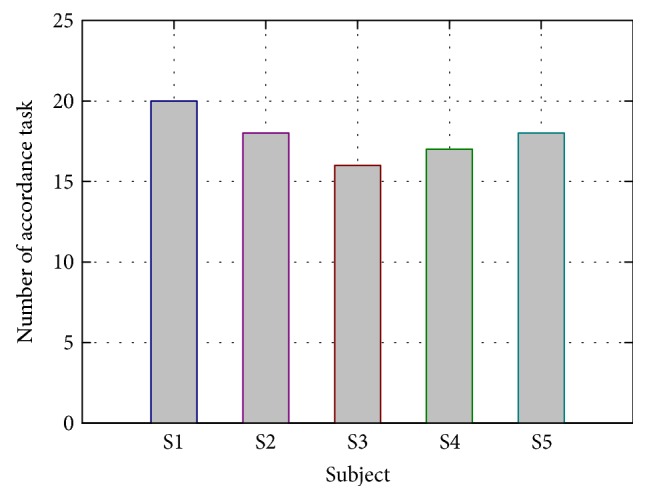
Number of accordance tasks for five subjects. A total of 20 tasks are set for subjects to trigger ankle robot based on clarification of SSVEP signals. Accordance task indicates that motion intention of subjects in that task is in accordance with actual stretching.

The results of combined ankle rotation tasks are shown in Table 1. All five subjects can trigger the ankle rehabilitation robot. Subject 1 conducts a total of 20 tasks without any discordance with his motion intentions, and subject 3 achieved 16 accordant tasks with the lowest success rate of 80% (Figure 5).

**Figure 6:**
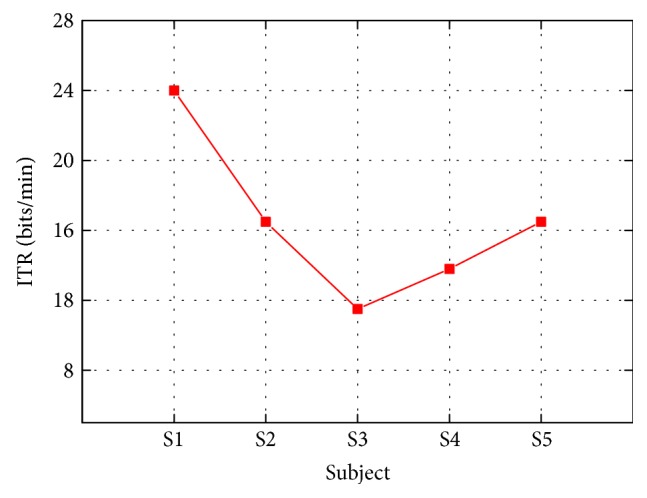
ITR for five subjects. The time applied to extract SSVEP signals is set as 5 seconds. A total of four targets are set in the SSVEP-triggered ankle training.

The biggest ITR of the robot for this training is set as 24 bits/min, and only subject 1 achieved it. The lowest ITR in this study is 11.5 bits/min when subject 3 achieved the success rate of 80% (Figure 6).

**Figure 7:**
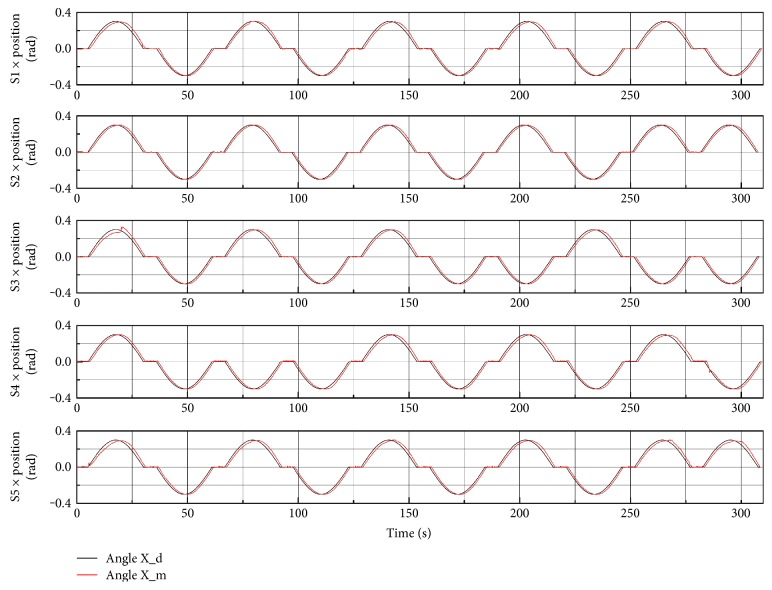
Tracking responses in joint space of DF/PF trajectory during ankle stretching conducted by five subjects. The red curve denotes actual moving trajectory, and the blue one represents the predefined ones. The amplitude of ankle rotation is 0.3 degree, and frequency is 0.02 Hz.

Tracking responses in joint space of combined DF/PF trajectory during ankle stretching are plotted in Figure 7. All subjects can trigger the robot to conduct the training. During conducting combined DF/PF tasks, subject 1 controls the training entirely following up his motion intention. Subjects 2, 4, and 5 have one inconsistent PF or DF training, and subject 3 has three inconsistent trainings.

## 4. Discussion

All five subjects succeeded in conducting training on the SSVEP-triggered robot following their motion intentions. The lowest success rate is 80%, and the highest one is 100%. The lowest ITR is 11.5 bits/min when the biggest one of the robots for this proposed training is set as 24 bits/min. The training is safe even when motion intentions of subjects are not consistent with real trajectories of ankle rotation.

This study introduces SSVEP signals to an ankle rehabilitation robot and combines partial characteristics of active ankle training with passive training. An information transmission loop between brains and ankle robots is proposed by combining SSVEP signals and virtual reality training circumstance on the ankle rehabilitation robot. This training strategy extends those active characteristics to subjects without ability to conduct active training. The feasibility study will be discussed from aspects of feasibility, motion intention-directed passive training, mechanism, and limitations.

### 4.1. Feasibility

In this study, five healthy subjects participated in the ankle training through gazing one of the four flickering sources to trigger passive ankle stretching on an ankle rehabilitation robot. One subject completely conducted the ankle training without any discordance between his motion intentions and actual rotation of the robot. Other subjects conducted the training with no less than 80% success rate. For subjects with weak motion ability of ankle joints, many of them still maintain a normal vision and can focus their attention on one of their interested things or objects over a period of time. Subjects with stroke even can achieve higher classification accuracy of extracting SSVEP signals from online experiments than normal subjects [28]. For subjects with weak motion ability of ankle joints, their confidence to recover health can be enhanced in further training when they actively conduct ankle training with 80% success rate through their own efforts, albeit with the assistance of a robot. Even if a judgment of the motion intention is wrong, the passive training will still be conducted following up the trajectory based on judgment of the robot. This definition can keep the integrity and continuity of the whole training and further improve efficiency of the training. During the training inconsistent with motion intention of subjects, ankle joints will still rotate following up trajectories of robot, which can maintain the whole quantity of ankle stretching motion.

The maximum ITR is 24 bits/min in this study, and the least ITR is 11.5 bits/min. Although the value of ITR is less than recordings in many other literature [27–29], it is suitable to apply in this study. Based on the definition of ITR expressed in (4), its value is computed based on the value of *T* and *N*, which is proportional to the value of *N*, and reversely proportional to the value of *T*. Although the ankle robot is designed with three rotational degrees of freedom, when combining the robot with virtual reality circumstance, the proposed SSVEP-based therapy retains two degrees of freedom; therefore, the value of *N* is set to 4. As mentioned in [11], in the passive training mode, the combined DF/PF trajectory is a sine wave with frequency of 0.02 Hz; therefore, the duration conducting single DF or PF trajectory will be approximately 25 seconds. In this study, it is suitable to set the value of *T* as 5 seconds, because subjects can have 5 seconds to prepare in mind to generate SSVEP signals, and at the same time, to accumulate energy in the body to conduct the judged training. Adding extra 5 seconds of motion intention judgment to 25 seconds of passive ankle training does not damage integrity of the whole training because its time compared to the total training is less. Subjects with weak motion ability of ankle joints might spend more than 5 seconds to prepare in their mind and accumulate energy in their body in advance before they can rotate ankles based on their own efforts. Therefore, extra 5 seconds of motion intention judgment applied to trigger passive training are comparable to their actual movement pattern of ankle joints.

### 4.2. Motion Intention-Directed Passive Training

For motion intention-directed passive training, virtual reality circumstance transfers rotation of ankle joints to trajectories of the cursor, which are requested to touch the target to complete the training [1, 13, 14]. Through observing the distance between the target and the cursor, human brains estimate which direction and how fast ankle joints should move and how much effort ankle joints should exert. Then, through neural pathways of the body, brains transfer the motion command to ankles. At the same time, trajectories of the cursor in virtual reality circumstance display the motion situation of ankle joints. After several loops of information transferring, the motion intention-directed passive training is accomplished when the target is reached by the cursor.

For pure passive training, subjects are requested to relax completely by tracking predefined trajectories of the robot [12]. Comparing with motion intention-directed passive training, the biggest difference of pure passive training is that brains of subjects might be in an idle state [11]. Relaxation of the whole body indicates that the brain does not response to any body motion. If their neural pathways are not blocked, internal feedback of ankle motion along nervous system should be maintained, which means that brains can feel the situation of ankle motion in their mind [17].

In this study, virtual reality circumstance is combined with passive training. Movement of the hammer on the rail can exactly display the rotation of the robot footplate. Therefore, subjects can get real-time visual feedback from movement of the hammer to know what trajectories ankle joints follow up and the real-time ankle position during the training. Once the hammer touches the targeted hamster, the hamster disappears immediately, and subjects can know that ankle joints have rotated to predefined maximum position. To summarize, the motion intention-directed passive training requires part of active engagement from subjects.

### 4.3. Mechanism

When subjects prepare or deliver ankle movements, EEG oscillatory activity at *α*-band (8–12 Hz) and *β*-band (12–30 Hz) decreases over the premotor and primary sensorimotor areas. This phenomenon is so called event-related desynchronization (ERD), representing increased activation of the corresponding cortical area. Event-related synchronization (ERS) is the phenomenon that power recovers to the resting condition at the end of movement [30]. Voluntary active movement, passive robot-assisted movement, and motor imagery all associate with ERD and ERS, and their oscillatory amplitude change is successively from big to small [31]. When a stroke patient was requested to repeatedly attempt DF of a paretic ankle joint at a comfortable pace, ERD-modulated functional electrical stimulation (FES) system could achieve short-term functional improvement comparing with FES alone [32]. To some extent, this supports the potential of the intention-directed passive training with respect to pure passive mode.

The proposed SSVEP-based training strategy extends partial characteristics of active training into passive training. Firstly, subjects with weak motion ability of ankle joints can actively trigger training. They can intentionally select certain training plan through gazing one of the flickering circles in virtual reality circumstance. Secondly, subjects with weak motion ability of ankle joints can pay more attention to their training. When in passive training, subjects are always requested to keep relax, and only follow up predefined trajectories of the robot [3, 10]. In general, the proposed intention-directed passive training strategy requires subjects to keep their attention on virtual reality circumstance to get visual feedback. In this way, subjects can not only know actual rotating position of ankle joints but also devote more efforts to the training.

### 4.4. Limitations

While preliminary experiments support the feasibility and promise of the proposed intention-based passive training on an ankle rehabilitation robot, this study still has some limitations. Firstly, EEG signal processing is performed simply through FFT which is a very basic algorithm. More advanced data process techniques should be involved to improve the reliability. Second, the proposed training strategy only involves very limited active engagement from participants. It can be a good idea to combine SSVEP signals with the whole training process for enhanced rehabilitation efficacy. The third one is that future experiments should recruit a large sample of patients with ankle disabilities. The question that how patients perform with such a training should be also investigated.

## 5. Conclusion

This study proposed a steady-state visual evoked potential- (SSVEP-) based passive training protocol on an ankle rehabilitation robot and validated its feasibility and promise on five healthy subjects. By combining SSVEP signals and the virtual reality circumstance, the ankle rehabilitation robot was found to be able to trigger the training based on their motion intentions. The virtual reality training circumstance also provides real-time visual feedback of ankle rotation. Experiments showed that subjects could succeed in conducting ankle training based on the SSVEP-triggered training strategy. Future work will focus on adopting more advanced data process techniques to improve the reliability of motion intention detection. The question that how patients perform with such a training strategy should be also investigated.

## Figures and Tables

**Table 1:** Result of conducting combined ankle movement tasks.

Subject	S1	S2	S3	S4	S5
Number of accordant task	20	18	16	17	18
Success rate	100%	90%	80%	85%	90%
ITR (bits/min)	24	16.5	11.5	13.8	16.5

*Note*. Two kinds of tasks, one for combined DF and PF training, and the other for combined INV and EV training. The total number of motion intentions requested to identify for every subject is 20. The duration for robot to judge the motion intention of subjects is 5 s. The number of flickering circles representing the motion intention of subjects was 4.
